# The ongoing value of first few X studies for COVID-19 in the Western Pacific Region

**DOI:** 10.5365/wpsar.2022.13.1.873

**Published:** 2022-03-24

**Authors:** Adrian J Marcato, James E Fielding, Kristy Crooks, Peter D Massey, Linh-Vi Le, Isabel Bergeri, Jodie McVernon

**Affiliations:** a The University of Melbourne, Melbourne, Victoria, Australia.; b Victorian Infectious Diseases Reference Laboratory, Peter Doherty Institute for Infection and Immunity, Melbourne, Victoria, Australia.; c Hunter New England Local Health District, Population Health, Wallsend, New South Wales, Australia.; d Menzies School of Health Research, Charles Darwin University, Darwin, Northern Territory, Australia.; e College of Medicine and Dentistry, James Cook University, Cairns, Queensland, Australia.; f Hunter New England Local Health District, Population Health, Tamworth, New South Wales, Australia.; g World Health Organization Regional Office for the Western Pacific, Manila, Philippines.; h World Health Organization, Geneva, Switzerland.; i Peter Doherty Institute for Infection and Immunity at the University of Melbourne and the Royal Melbourne Hospital, Melbourne, Victoria, Australia.; j Murdoch Children’s Research Institute, Melbourne, Victoria, Australia.

Studies of the first few “X” (FFX) – formerly known as “First Few 100” – cases involve rapid collection of data and specimens from the cases of a novel pathogen or emerging variants and their close contacts. Collection of standardized high-quality clinical, epidemiological, virological and serological data in FFX studies can provide insight into transmission dynamics, severity, risk factors for severe disease and the clinical spectrum of disease. These data can be used in risk assessment and modelling studies, to forecast potential impact and guide preparedness planning and public health interventions.

Independent studies of coronavirus disease 2019 (COVID-19), caused by severe acute respiratory syndrome coronavirus 2 (SARS-CoV-2), have provided insights into key transmissibility and severity parameters. (*1*) Although these studies are valuable in contributing to the growing body of scientific evidence on COVID-19 epidemiology, there is need for a greater number of harmonized studies (e.g. FFX studies) that can be rapidly implemented in early epidemic phases. (*2*)

In early January 2020, the World Health Organization (WHO) adapted and added to existing pandemic influenza and Middle East respiratory syndrome coronavirus (MERS-CoV) protocols for COVID-19 and rebranded them as UNITY studies – a global sero-epidemiological standardization initiative. UNITY protocols aim to increase evidence-based knowledge for action, and are an invaluable tool for improving equity by providing harmonized and fit-for-purpose protocols for all income and resource settings. (*3*) UNITY studies allow for timely comparison and aggregate analysis of data across countries and regions, to contextualize data to different settings and offer a depth of understanding that is not readily available using other platforms.

WHO solicited interest in implementing these protocols during the COVID-19 pandemic from partners to address knowledge gaps and inform public health response measures. (*4*) Despite being in an unprecedented pandemic, many countries were able to leverage existing infrastructure to implement UNITY studies. Insights from participating countries are centrally reported to WHO headquarters and regional offices, and include contributions from 98 WHO Member States (including Australia, Mongolia, the Philippines and Singapore in the Western Pacific Region). (*4*, *5*)

More in-depth understanding of the epidemiology of COVID-19 gained through such studies can be used to inform adaptive and ongoing control strategies. For example, in early 2020, a study aligned with FFX and UNITY conducted in China showed that most secondary cases were probably infected around the time of symptom onset of the primary cases. (*6*) This highlighted the need for household infection control measures, given that immediate intervention by local health authorities following symptom onset of the primary case is difficult to achieve. Data from another aligned study conducted in the United Kingdom of Great Britain and Northern Ireland (United Kingdom) established a sensitive and specific symptom profile of COVID-19, including the reporting of anosmia in patients. This symptom was later added to the United Kingdom’s COVID-19 symptom list. (*7*) There is also continuing uncertainty about the role of children in spreading COVID-19 and the extent of true asymptomatic and pre-symptomatic transmission. Although the spread appears to be influenced by social settings and household structures, public health interventions (e.g. test, trace and isolate; spontaneous and imposed behavioural and distancing measures and mobility restrictions; communication campaigns; and varying degrees of community engagement and cohesion in response) have led to differing rates of transmission within and between countries. FFX studies can provide opportunities to explore transmission dynamics and severity during all epidemic phases, provided that contacts of cases can still be traced.

FFX and other UNITY studies are well placed to provide information on SARS-CoV-2 variants such as α, β and delta, which are marked by different biological characteristics to those previously observed in epidemiological studies. (*8*) Pooling of data from FFX studies may help us to understand how SARS-CoV-2 could behave in the Western Pacific, particularly in settings that have not yet experienced uncontrolled epidemics and in populations with low vaccination coverage or low levels of natural immunity. Areas with limited resources to conduct intensive surveillance studies would benefit from globally standardized data collection and analysis to assist with more nuanced planning for future outbreaks.

FFX studies provide a platform to compare epidemiology between waves and jurisdictions and can be used to inform targeted and context-specific public health interventions. For example, Australia and Singapore – countries in the Western Pacific – experienced subsequent waves of epidemic activity that exhibited different epidemiological patterns to earlier waves. The first wave in Australia predominantly featured cases acquired overseas or their close contacts, whereas the second wave was amplified in aged-care and health-care setting outbreaks that led to community transmission. (*9*) Singapore’s initial epidemic was characterized by outbreaks in migrant workers residing in dormitories, with low-level community transmission. In late 2021, Singapore experienced an epidemic wave of the delta variant with widespread community transmission. (*10*) In both settings, culturally and linguistically diverse communities and workers who were unable to work from home have been disproportionately infected. (*9*, *10*)

Existing FFX study populations can be expanded into longitudinal cohorts with extended follow-up to address questions regarding persistence of immunity following both natural and vaccine-induced immunity, and its effectiveness in preventing infection and disease upon re-exposure. These data will be critical in informing future control measures, particularly with the emergence of new variants and the commencement of vaccination campaigns.

For maximum utility, countries should exercise or pilot these studies in advance of future outbreaks – for example, at the beginning of influenza seasons – to facilitate timely implementation during emergencies. Piloting will allow countries to consider data collection and management, laboratory testing and capacity, ethics and governance approvals, identify a suitable workforce and develop workflows in advance of outbreaks, to ensure that they are effective.

Piloting should also consider developing scalable, feasible and culturally appropriate methods for collecting data and specimens, to improve equity and health outcomes for the vulnerable and those at greater risk of disease. These methods should be developed with communities for communities. (*11*) Historical evidence shows that previous pandemics have disproportionately impacted First Nations peoples. (*12*-*15*) Adapting FFX and UNITY studies within a First Nations context can lead to a deeper understanding of the experience of families, explore household transmission in different types of households and improve understanding of how studies can be operationalized to inform culturally appropriate and safe disease control strategies.

FFX studies and the broader suite of UNITY studies remain incredibly useful in the current regional and global context, and they could provide ongoing robust and comparable evidence of COVID-19 epidemiology in low- and middle-income countries as the pandemic evolves. Investing in UNITY studies, readiness and preparedness planning will better support the ongoing COVID-19 response and help to ensure research equity and improve capacity to respond rapidly to future emerging pathogens. Pandemic-ready, flexible systems are paramount to support an equitable, proportionate and informed emergency public health response.
